# Surveying patients to assess timeliness of cancer diagnosis

**DOI:** 10.1515/dx-2024-0203

**Published:** 2025-10-27

**Authors:** Claire E. O’Hanlon, Carl Berdahl, Feifei Ye, Elie Ohana, Anagha Tolpadi, Melissa A. Bradley, Elizabeth Marsolais, Maxim Ptacek, Andrew J. Henreid, Rebecca Anhang Price

**Affiliations:** RAND, Santa Monica, CA, USA; Cedars-Sinai Medical Center, Los Angeles, CA, USA; RAND, Pittsburgh, PA, USA; RAND, Arlington, VA, USA; University of California – Los Angeles, Los Angeles, CA, USA; University of Connecticut, Storrs, CT, USA

**Keywords:** cancer, delayed diagnosis, delay, quality measurement, quality improvement, patient experience

## Abstract

**Objectives:**

To develop and test feasibility of patient survey-based quality measures to assess timeliness of cancer diagnosis.

**Methods:**

We developed a 39-item survey through review of literature and measures, input from experts, and cognitive testing. We field-tested it in an urban health system among patients with new cancer diagnoses (prior 3–9 months); surveys were administered by web and mail September–October 2023. We calculated top-box scores and conducted confirmatory factor analysis for evaluative items and assessed construct validity through correlations to the diagnostic interval, number of health care visits, and overall assessments of care quality.

**Results:**

The overall response rate was 23.9 %. The 276 respondents primarily spoke English (89 %), were non-Hispanic white (66 %), age 65+ (68 %), and college graduates (67 %). More than a quarter believed their cancer could have been diagnosed much sooner. Better communication with health care professionals, timely receipt of usual and specialty care, and ease of receipt and follow-up of tests, X-rays, and scans were positively associated with patients reporting that health care professionals could not have diagnosed the cancer much sooner. Patients reporting it was always easy to receive needed tests, X-rays, and scans and that they always received follow-up results were more than three times more likely to report a diagnostic interval less than or equal to 30 days (p<0.001).

**Conclusions:**

Patients can report on barriers to timely diagnosis associated with the length of their cancer diagnostic interval. Surveys are a promising source of information regarding opportunities to improve timeliness of cancer diagnosis.

## Introduction

Timely diagnosis is a critical component of high-quality cancer care [1], [2], [3]. Timely cancer diagnosis may result in improved survival, diagnosis at earlier stages, and improved quality of life [4]. Measuring timeliness and delays in cancer diagnosis is complex [5], and proposed definitions vary [6], 7]. Measurements of timeliness can be operationalized as a continuous length of time, a binary judgment of timeliness, or the occurrence of events or experiences associated with delays to timely medical care. Some factors contributing to delays are not unique to cancer care, including difficulty accessing care (navigation difficulties, wait times for appointments, or insurance barriers), poor communication with clinicians, and not getting appropriate imaging or other tests and referrals to specialists. However, all these measurement approaches present implementation challenges in the fragmented US health care system, as patients may receive care from multiple clinicians and facilities on their journey to cancer diagnosis.

Eliciting patient perspectives is the only way to understand certain aspects of care quality, such as the effectiveness of clinician communication. Surveying patients is also an important way of understanding breakdowns in complex diagnosis processes, which are particularly difficult to study in the US context, as clinicians and facilities rarely share electronic health records (EHRs) and patients often have multiple payers, complicating analyses of EHR and claims. Patient reports and experiences have been a critical tool for improving diagnosis [8], [9], [10], [11], and patient experience surveys are an important method for understanding and measuring health care quality [12]. Patient experience may be especially important for understanding cancer diagnosis, which can be a prolonged, complex, and multi-step process [5]. Surprisingly, little is known about patient experiences of cancer diagnosis in the United States (US), as most of this research has been conducted in other countries [13], [14], [15], especially the United Kingdom (UK) [16], [17], [18], [19]. Large scale patient experience surveys in the US, such as the US Consumer Assessment of Healthcare Providers and Systems (CAHPS^®^) Cancer Care surveys, focus on assessing treatment and not diagnosis [20]. In this study, we aimed to develop and test the feasibility of administering survey-based patient experience measures of timeliness of cancer diagnosis. We report results on patient experiences as well as psychometric properties of the measures.

## Methods

We complied with all relevant national human subjects regulations, institutional policies, and tenets of the Helsinki Declaration. This research was approved by the RAND Human Subjects Protection Committee and Cedars-Sinai Institutional Review Board.

### Survey development

In keeping with best practices for patient experience survey development [21], we conducted an environmental scan; sought input from technical experts, stakeholders, and potential users; drafted and iterated on the survey; and conducted cognitive interviews prior to field-testing.

#### Environmental scan

We reviewed the literature on timeliness and delays in cancer diagnosis and identified the closest existing survey questions and measures. We also interviewed 21 subject matter experts in diagnosis, quality measurement, cancer, and patient surveys. Questions included perspectives on timeliness of cancer diagnosis as a quality gap, causes and factors associated with missed or delayed cancer diagnoses, and usefulness and challenges of surveying patients on cancer diagnosis experiences.

#### Expert input

We convened a 14-member Technical Expert/Clinical User/Patient Panel (TECUPP), which included seven experts in clinical medicine, quality measurement, and diagnosis alongside seven patients or care partners with lived cancer experience. The TECUPP met in early 2023 to discuss potential survey and measure domains using an established patient-centered framework [22] and iterate on the draft survey. TECUPP members were asked to discuss which factors were (a) important for understanding timeliness of cancer diagnosis, (b) observable to patients and/or care partners, and (c) actionable for health care organizations.

#### Draft survey

Based on the environmental scan and feedback from the TECUPP, we developed a draft survey. We used or adapted existing items when available [13], [23], [24], [25] and created *de novo* items when they did not.

#### Cognitive interviews

To test the degree to which potential survey respondents consistently and accurately comprehended, interpreted, and answered draft questions, we conducted cognitive interviews with 15 patients (identified using a different sampling procedure from the survey) with lived experience of cancer diagnosis within two years and a variety of cancer types and educational levels. We revised the draft survey to reflect feedback from interviewees.

#### Final survey

The final version of the “Survey About Care Leading Up to Cancer Diagnosis” (Supplement) includes 29 questions related to timeliness of cancer diagnosis, including self-reported cancer type, stage, and route to diagnosis (symptoms, screening, or incidental findings); key dates (first symptoms or test results, diagnosis, referral to specialist); source and involvement of usual care; referrals to specialists; barriers to seeing usual and specialty providers; communication and respect from usual and specialty providers; access and follow-up to test results; access to interpretation; and global experience ratings. We considered 12 items on patients’ perception of getting timely care, communication and respect from providers, access and follow-up to test results, and global experience ratings as “evaluative” items potentially suitable for measure development. In addition, the survey includes eight questions about the demographics of the patient and proxy respondent (if applicable) as well as two open-ended questions for patients to provide additional information about what went well or poorly in their experiences of cancer diagnosis.

### Survey sample

We identified survey-eligible patients through EHR at Cedars-Sinai, a large integrated health system serving a diverse population in urban Los Angeles. Eligible patients had a cancer ICD-10 encounter diagnosis code in the last 3–9 months (December 2022 to June 2023) and no encounters with a cancer ICD-10 code within five years prior. All cancer ICD-10 codes (C-codes) were included, except common skin cancers (C44) and cancer-like syndromes (C96). Patients were determined to be ineligible and were excluded from further analysis following survey administration if they disavowed being diagnosed with cancer in the last 12 months (n=19) or skipped all evaluative items assessing perceived access and quality of care (n=3).

### Data collection

Survey data were collected via a web survey with mail survey follow-up. Patients with an email address on file (n=1,102) were sent an email with a survey link and a reminder email two days later. Patients who did not complete the web survey or who did not have an email address on file were sent a paper packet containing the survey and a business reply envelope one week after the initial survey email. Patients who had not completed the web or mail survey after three weeks were mailed a second paper packet. The data collection period was closed three weeks after the second packet was mailed. All survey and recruitment materials were in English.

### Analyses

#### Descriptive analyses

We calculated the diagnostic interval starting with the patient-reported date of first visit/test leading to their cancer diagnosis and ending with the date a health care professional communicated the diagnosis. If month and/or year were not provided, we considered the diagnostic interval to be missing. If the first visit/test and diagnosis date were reported to be the same month and year, but no day was provided, we estimated the diagnostic interval to be 15 days.

To promote ease of interpretation and set high standards for care quality, we calculated top-box scores for each evaluative survey item [26]. This means we classified response(s) indicating the best experiences as 100 and all other responses as 0 (e.g., ‘‘always’’ = 100; all other responses = 0; rating of 9 or 10 out of 10=100; all other responses = 0). We report stratified results by initial presentation and cancer stage at diagnosis.

#### Measure properties

Among the 12 evaluative items on the survey, two items were considered as single-item measures of patients’ overall assessments of care (Overall Rating of Care and No Perceived Delay in Cancer Diagnosis). We conducted confirmatory factor analysis (CFA) to evaluate the factorial structure of the remaining 10 evaluative items assessing various aspects of care. We hypothesized two single-item measures (Getting Timely Usual Care and Getting Timely Specialty Care, measured by two items about how often patients were able to see usual or specialty health care professional(s) as soon as needed) and three factors: Communication with Usual Care Professionals (3 items), Communication with Specialists (3 items), and Getting Tests and Results (2 items). We used means- and variance-adjusted weighted least squares to account for the dichotomous nature of top-box scores. We used a criterion of factor loadings ≥ 0.40 [27] for inclusion within proposed factors and assessed overall model fit using the Comparative Fit Index (CFI), the root mean square error of approximation (RMSEA), and weighted root mean square residual (WRMR) (models with good fit typically have CFI>0.95, RMSEA<0.05, and WRMR<1.0 [28], 29]). With the factorial structure confirmed, we calculated composite measure scores for each factor as the average of top-box-scored items included in that factor and assessed the degree to which the composite measures assess distinct content domains by calculating their correlations. Correlations exceeding 0.80 suggest insufficiently distinct aspects of care. We also calculated the composite reliability (the reliability of observed scores calculated as composites [e.g. mean] of individual items) using coefficient Omega, which uses parameters in the factor-analytic model and is recommended over Cronbach’s alpha [30], 31]. We calculated categorical Omega to account for the ordinal nature of item top-box scores [32], 33]. Larger values indicate more precise measurement of the underlying construct. Composite reliability of 0.70 or higher is considered adequate [34].

To assess construct validity, we examined the degree to which the composite measures were related to overall assessments of care by running linear or logistic regression models in which each measure predicted the following outcomes calculated from survey responses: Overall Rating of Care, Perceived Timeliness of Cancer Diagnosis (i.e., no perceived delay), Number of Visits to Health Care Professional During Diagnostic Interval, and Diagnostic Interval less than or equal to 30 days. A diagnostic interval of one month is commonly used in studies of cancer diagnostic intervals and aligns with international benchmarks, such as the National Health Service’s Faster Diagnosis Standard [35].

## Results

### Survey results

We received 276 surveys from eligible respondents for an overall response rate of 23.5 %; 21.4 % responded by web and 78.6 % by mail. Approximately two-thirds were age 65 or older; 6.5 % were non-Hispanic Black or African American, 12.3 % were Hispanic, and 65.6 % were non-Hispanic white (Table 1). Two-thirds of respondents had a college degree or more. 11 percent of respondents were proxies responding on behalf of the patient. Respondents had similar sex and ethnicity breakdowns compared to the overall sample individuals to whom we sent the survey (Supplementary Appendix Table A.1); respondents were less likely to be Black/African-American and were generally older than the overall sample.

**Table 1:** Characteristics of survey respondents (n=276).

Characteristic	n (%)
**Sociodemographic characteristics**	

Sex	
Female	151 (54.7 %)
Male	125 (45.3 %)
Age	
18–39 years	12 (4.4 %)
40–64 years	76 (27.7 %)
65–79 years	137 (50.0 %)
>80 years	49 (17.9 %)
Race/ethnicity	
Non-hispanic black or African American	18 (6.5 %)
Hispanic (of any race)	34 (12.3 %)
Non-hispanic white	181 (65.6 %)
Other	43 (15.6 %)
Education	
Less than high school	7 (2.5 %)
High school graduate or GED	14 (5.1 %)
Some college or 2-year degree	71 (25.8 %)
College degree or more	183 (66.5 %)
Primary language^a^	
English	239 (88.8 %)
Spanish	8 (3.0 %)
Other	22 (8.2 %)

**Clinical characteristics**	

Cancer type	
Anal	6 (2.2 %)
Bladder	8 (2.9 %)
Brain	8 (2.9 %)
Breast	72 (26.2 %)
Cervical	3 (1.1 %)
Colon/rectal	12 (4.4 %)
Endometrial/uterine	9 (3.3 %)
Head and neck	7 (2.5 %)
Kidney	5 (1.8 %)
Leukemia/lymphoma/myeloma	28 (10.2 %)
Liver	6 (2.2 %)
Lung	22 (8.0 %)
Melanoma	5 (1.8 %)
Ovarian	6 (2.2 %)
Pancreatic	18 (6.5 %)
Prostate	36 (13.1 %)
Stomach/esophageal	7 (2.5 %)
Thyroid	6 (2.2 %)
Other	11 (4.0 %)
Stage at diagnosis	
Stage 0 (*in situ*)	15 (5.7 %)
Stage 1	63 (23.9 %)
Stage 2	39 (14.8 %)
Stage 3	28 (10.6 %)
Stage 4/metastatic	41 (15.5 %)
Don’t know/not sure	78 (29.5 %)
Initial presentation	
Screening test abnormal	60 (21.9 %)
Incidental finding	84 (30.7 %)
Symptoms	122 (44.5 %)
Diagnostic interval	
≤30 days	129 (46.7 %)
31–60 days	50 (18.1 %)
61–90 days	22 (8.0 %)
91–180 days	22 (8.0 %)
181+ days	23 (8.3 %)
Missing	30 (10.9 %)
Number of visits to health care professional during diagnostic interval	
0–1	61 (22.7 %)
2	66 (24.5 %)
3–4	78 (29.0 %)
5+	56 (20.8 %)
Don’t know/not sure	8 (3.0 %)

All characteristics self-reported in survey response data. Categories not adding up to 276 indicate missing. Missing shown when >5 %. ^a^Survey materials were only available in English. Although 30 respondents reported primary languages other than English, we received English-language survey responses from all of these respondents. Of the respondents, only 13 reported ever wanting interpreter services during their cancer diagnosis, indicating that most of these individuals were comfortable communicating in English even if it was not their primary language. Five respondents reported using the assistance of someone to translate the survey.

Respondents were diverse regarding cancer type, stage at diagnosis, and initial presentation. The most common cancer types were breast; prostate; leukemia, lymphoma, or myeloma; lung; and pancreatic (Table 1). Compared to national incidence rates, breast cancer was overrepresented among respondents, while prostate, lung, and colorectal cancers were each slightly underrepresented [36]. While 29.5 % of respondents reported not knowing or being unsure of their cancer stage, 29.6 % reported being diagnosed at Stage 0 or 1, 25.4 % at 2 or 3, and 15.5 % at Stage 4. Forty-five percent reported their diagnostic interval began with a visit to a health care professional to discuss symptoms; 30.7 % reported their cancer diagnosis initially presented as an incidental finding and 21.9 % reported their diagnostic interval began with an abnormal result on a cancer screening test.

Patient-reported diagnostic intervals (days between initial presentation and diagnosis) varied; 46.7 % of patients reported intervals of 30 days or fewer and 8.3 % reported intervals greater than 180 days; 10.9 % could not provide sufficient information to place the respondent in an interval category. Only 3.0 % of patients said they were unsure of number of visits to a health care professional during the diagnostic interval, while 22.7 % reported no visits or one visit and 20.8 % reported having five or more visits. Patients who were diagnosed at later stages and had symptomatic initial presentations tended to report shorter diagnostic intervals (Table 2).

**Table 2:** Length of diagnostic interval and number of visits to health care professionals during diagnostic intervals; overall and for subgroups of interest.

		Initial presentation (n=266)				Cancer stage at diagnosis (n=186)			
	All	Screening test (n=60)	Incidental finding (n=84)	Symptomatic (n=122)	p-Value	0–1	2–3	4	p-Value
	(n=276)					(n=78)	(n=67)	(n=41)	
Diagnostic interval (continuous)									
Mean, days^a^	69	79	93	48	0.059	92	69	44	0.188
Diagnostic interval (binary)									
≤30 days	52 % (129/246)	42 % (24/57)	45 % (34/75)	63 % (66/105)	0.014	45 % (33/74)	52 % (32/62)	76 % (28/37)	0.008
≤90 days	82 % (201/246)	79 % (45/57)	77 % (58/75)	87 % (91/105)	0.224	73 % (54/74)	81 % (50/62)	89 % (33/37)	0.131
Number of visits during diagnostic interval (categorical)									
0–1 visits	23 % (61/261)	21 % (12/56)	20 % (16/80)	27 % (31/116)	0.937	15 % (11/72)	30 % (20/67)	28 % (11/39)	0.092
2 visits	25 % (66/261)	27 % (15/56)	28 % (22/80)	23 % (27/116)		28 % (20/72)	21 % (14/67)	15 % (6/39)	
3–4 visits	30 % (78/261)	30 % (17/56)	29 % (23/80)	30 % (35/116)		39 % (28/72)	25 % (17/67)	23 % (9/39)	
5 or more visits	21 % (56/261)	21 % (12/56)	24 % (19/80)	20 % (23/116)		18 % (13/72)	24 % (16/67)	33 % (13/39)	

We tested statistical significance of group comparison for initial presentation and cancer stage diagnosis using ANOVA tests for diagnostic interval (continuous), and Chi-square tests for other variables.

### Quality of care measure properties

We derived seven quality of care measures from the survey, including two single-item overall assessments of care, and five measures assessing aspects of care quality during the cancer diagnostic interval (two single-item measures, Getting Timely Usual Care and Getting Timely Specialty Care, and three multi-item, composite measures, Communication with Usual Care Professionals, Communication with Specialists, and Getting Tests and Results, which were confirmed in CFA). The CFA model (two single item measures and three factors representing three multi-item composite measures) provides an excellent fit to the data, χ^2^_(27)_=26.18, p=0.51; CFI=1.00; RMSEA=0.00; WRMR=0.49. Table 3 displays the factor loadings and corrected item-total correlation for the eight evaluative items proposed for three factors, along with internal consistency reliability (categorical Omega) for each composite measure. The factor loadings range between 0.71 and 1.00 and corrected item-total correlations range between 0.39 and 0.76, suggesting these items are strong indicators of the corresponding factor. Top-box-scored items included in each factor were averaged to generate scores for each composite measure. The five measures assessing aspects of care quality are moderately correlated, with a range of r=0.25 to r=0.50 (Table 4), indicating that they measure related but distinct aspects of care. Measures that are least correlated are Getting Timely Usual Care and Communication with Specialists (0.25) and Getting Timely Usual Care and Getting Tests and Results (r=0.31).

**Table 3:** Psychometric properties of proposed composite measures assessing timeliness of cancer diagnosis.

Composite measures and component items	Item-total correlation	Factor loading	Top-box score
Communication with Usual Care Professionals During the time leading up to your cancer diagnosis, how often did your usual health care professional (s)	Categorical omega = 0.86, 95 % CI [0.80, 0.91]		76 % (149.5/197)
1. Listen carefully to you?	0.71	0.92	72 % (138/193)
2. Take your concerns seriously?	0.75	1.00	73 % (141/192)
3. Treat you with dignity and respect?	0.58	0.89	85 % (164/192)
Communication with Specialists During the time leading up to your cancer diagnosis, how often did the specialist(s) you saw	Categorical omega = 0.88, 95 % CI [0.79, 0.93]		88 % (167.83/191)
1. Listen carefully to you?	0.70	0.96	85 % (162/190)
2. Take your concerns seriously?	0.76	0.97	86 % (164/190)
3. Treat you with dignity and respect?	0.63	0.91	92 % (176/191)
Getting Tests and Results During the time leading up to your cancer diagnosis	Categorical omega = 0.74, 95 % CI [0.67, 0.80]		67 % (182/272)
1. How often was it easy to get the tests, X-rays, or scans you needed?	0.39	0.84	63 % (168/267)
2. When someone ordered a test, X-ray, or scan for you, how often did someone follow up to give you those results?	0.39	0.71	72 % (191/267)

CI, confidence interval.

**Table 4:** Correlations among composite measures and single-item measures.

	Getting Timely Usual Care	Communication with Usual Care Professionals	Getting Timely Specialty Care	Communication with Specialists	Getting Tests and Results
Getting Timely Usual Care: during the time leading up to your cancer diagnosis, how often were you able to see your usual health care professional(s) as soon as you needed?	–				
Communication with Usual Care Professionals^a^	0.42	–			
Getting Timely Specialty Care: during the time leading up to your cancer diagnosis, how often were you able to see the specialist(s) as soon as you needed?	0.41	0.50	–		
Communication with Specialists^a^	0.25	0.43	0.45	–	
Getting Tests and Results^a^	0.31	0.41	0.45	0.47	–

Correlations presented are Pearson correlations. All correlations are significant at p<0.001. ^a^Item text shown in Table 3.

#### Overall reported care experiences

**Figure 1:**
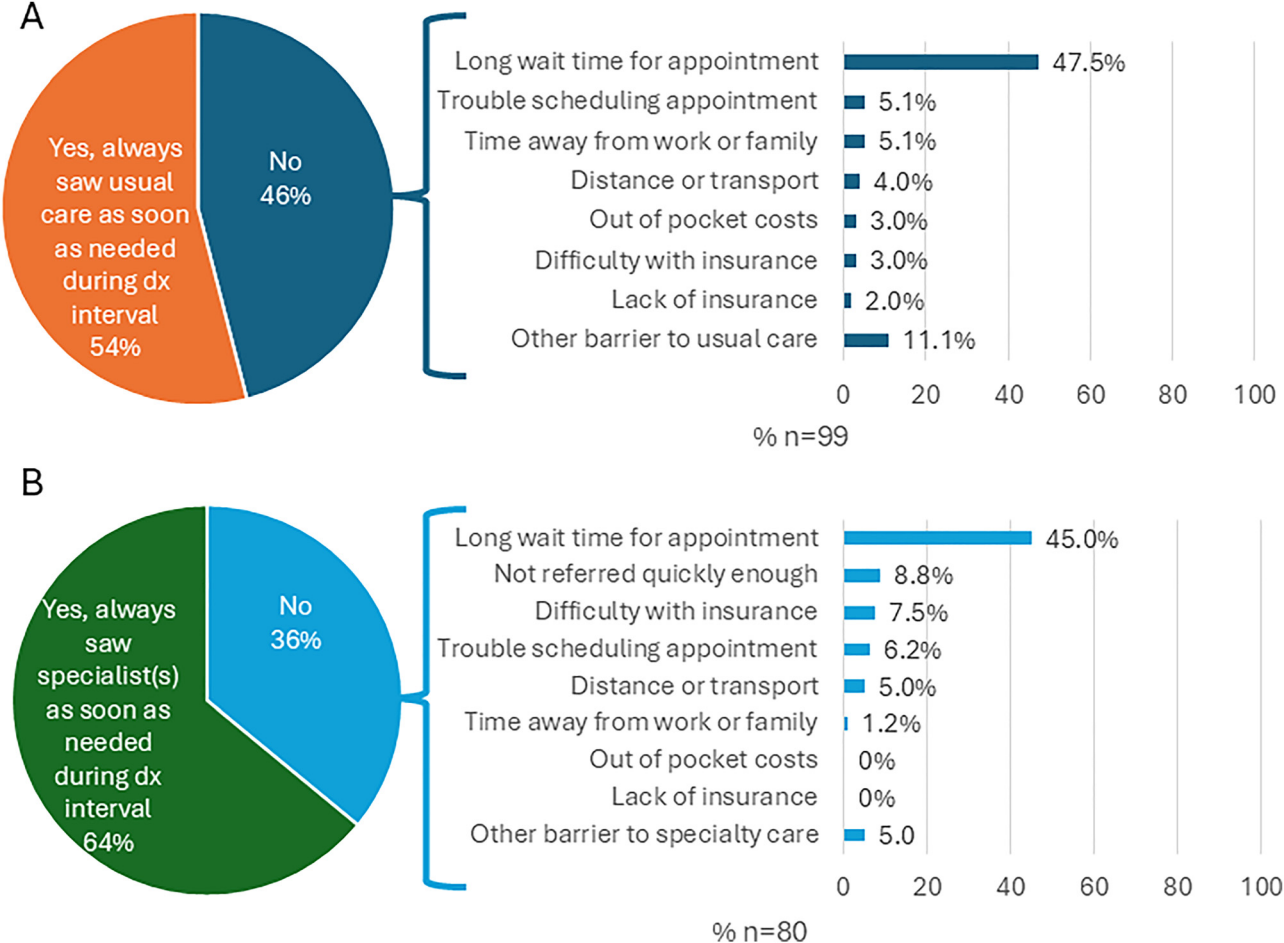
Descriptive results of access and barriers for usual care (A) and specialty care (B).

Distributions of responses for each measure are shown in Supplementary Appendix Table A.2. Approximately four out of five (79 %) respondents reported having a usual source of care prior to the diagnostic interval, and 90 % of these respondents talked to their usual care provider during the diagnostic interval. Half (46 %) reported they did not “always” see their usual care provider as soon as needed during the diagnostic interval (Figure 1A); the predominant barrier to usual care reported was long wait time for an appointment (48 %).

Of patients who saw their usual care providers during the diagnostic interval, 70 % reported their usual care provider referred them to a specialist. Whether or not they were referred, 69 % percent of respondents reported seeing a specialist during the diagnostic interval. Of these, 36 % reported they did not “always” see the specialist as soon as needed during the diagnostic interval (Figure 1B). The predominate barrier to specialty care reported was also long wait time for an appointment (45 %), although not being referred quickly enough and difficulty with insurance coverage were also noted by 8.8% and 7.5 % of respondents, respectively.

Respondents reported favorable experiences overall (Table 3 and Supplementary Appendix Table A.3). Patients reported better communication with specialists than with usual care providers; top-box scores for Communication with Usual Care Professionals and Communication with Specialists composite measures were 76 and 88, respectively. The overall top-box score for the Getting Tests and Results composite measure was somewhat lower (67). Nearly three-quarters (74 %) of respondents rated their overall care during the diagnostic phase a 9 or 10 out of 10, while 10 % rated it 6 or less. Nearly two-thirds of respondents (64 %) believed their cancer could not have been diagnosed much sooner.

**Figure 2:**
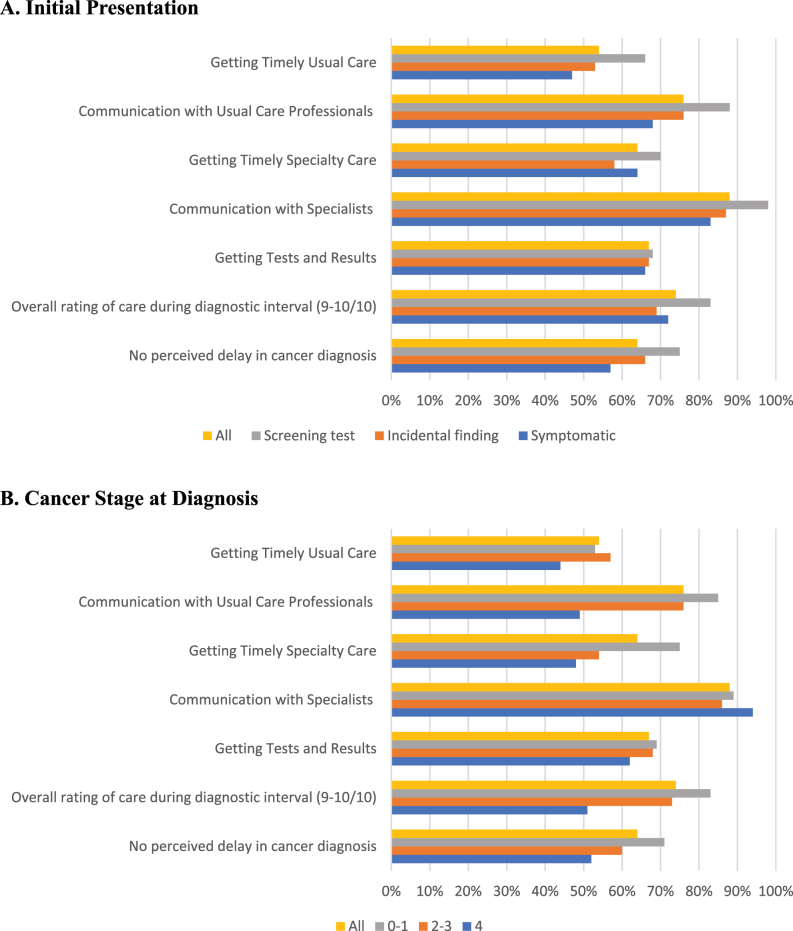
Top-box scores for timeliness of care leading up to cancer diagnosis measures by (A) initial presentation (B) cancer stage at diagnosis. Top-box scores classify the response(s) indicating the best experiences as 100 and all other responses as 0 (e.g., ‘‘always’’ = 100; all other responses = 0; rating of 9 or 10 out of 10=100; all other responses = 0).

Patients who were diagnosed at later stages and had symptomatic initial presentations tended to report overall worse care experiences, though this was not true in all cases. Exceptions included patients with symptomatic initial presentations reporting higher rates of results follow-up of tests, X-rays, and scans (Figure 2A), and patients diagnosed at Stage 4 reporting better communication with specialists and results follow-up of tests, X-rays, and scans than patients diagnosed at earlier stages (Figure 2B).

#### Association between measures and perceived timeliness of diagnosis, diagnostic interval, and overall ratings of care

All measures had strong, statistically significant associations with perceived timeliness of cancer diagnosis and overall ratings of care (Table 5); Communication with Usual Health Care Professionals, Communication with Specialists, and Getting Tests and Results were the strongest predictors of these outcomes. Getting Tests and Results, and Getting Timely Specialty Care were the strongest predictors of a diagnostic interval less than or equal to 30 days. In models that included all measures simultaneously (rather than individually), the strongest predictors of outcomes remained the same, although coefficients and odds ratios were somewhat smaller (Supplementary Appendix Table A.4). Measures were mostly not associated with the number of visits to a health care professional during the diagnostic interval.

**Table 5:** Association between quality measure top-box scores and overall ratings of care, number of visits during diagnostic interval, diagnostic interval less than days, and perceived timeliness of cancer diagnosis.

	Perceived timeliness of cancer diagnosis	Diagnostic interval ≤30 days	Overall rating (higher is better)	Number of visits during diagnostic interval
**Models assessing measures one-by-one**	**Odds ratio [95 % CI]**		**Unstandardized regression coefficients [95 % CI]**	

Getting Timely Usual Care	3.37^c^ [1.70, 6.69]	1.41 [0.77, 2.57]	1.33^c^ [0.78, 1.89]	0.13 [−0.19, 0.45]
Communication with Usual Care Professionals	14.07^c^ [4.95, 39.98]	1.10 [0.50, 2.43]	3.20^c^ [2.56, 3.83]	−0.37 [−0.80, 0.06]
Getting Timely Specialty Care	3.63^c^ [1.74, 7.57]	2.10^a^ [1.09, 4.03]	1.58^c^ [1.12, 2.03]	−0.14 [−0.47, 0.19]
Communication with Specialists	10.93^c^ [2.90, 41.18]	1.71 [0.55, 5.35]	3.17^c^ [2.44, 3.91]	−0.51 [−1.05, 0.04]
Getting Tests and Results	6.63^c^ [3.12, 14.09]	3.28^c^ [1.68, 6.41]	2.40^c^ [1.88, 2.92]	−0.11 [−0.47, 0.25]

^a^p<0.05; ^b^p<0.01; ^c^p<0.001.

## Discussion

In our study of patients recently diagnosed with cancer in a large integrated health system, patients reported barriers to timely diagnosis that were both associated with the cancer diagnostic interval and actionable for health care providers and systems. Patient reports of timely access to specialty care and ease of getting tests and results were strongly associated with shorter diagnostic intervals.

Our results demonstrate that patient-reported measures can provide important information about diagnosis experiences that may not be captured by other data sources. For example, respondents who reported that they experienced barriers to accessing usual care and specialty care, long wait times for appointments was by far the most frequently reported barrier. Patient ratings of timeliness have been shown to be correlated with real appointment wait times [37]. Long wait times are potentially actionable by health care systems [38], 39].

Most respondents (90 %) were able to report the dates of both their first visit/test and their diagnosis to at least the month and year. Previous work has found concordance of patient reports of this information with administrative data [40]. Wide variation was reported in the number of visits during the diagnostic interval; about half of respondents reported three or more visits. Previous work has found that only 23 % of UK cancer patients experienced three or more consultations before referral for cancer [41], though the contextual differences of the UK’s general practitioner gatekeeper model make these numbers hard to compare. About 28 % of patients believed their cancer could have been diagnosed much sooner, which is similar to rates of previously reported rates as assessed by clinicians (24 %–34 %) [42], 43].

This work has several limitations. This study was conducted among patients receiving cancer care in a single, large integrated health system highly ranked for health care quality and serves patients with high levels of education and insurance coverage. To ensure sufficient sample size for analysis of patient experience measures, we analyze all cancer types together, even though they may have different acceptable diagnostic intervals. The survey materials were in English only and most respondents primarily spoke English, which limits our ability to understand the role of language in timeliness of cancer diagnosis [44]. About one-quarter of the sample responded to the survey, comparable to other patient experience surveys in broad national use [45]. However, results still represent a minority of potential respondents and our sample was small overall. These issues may reduce generalizability of both the results and measure performance.

We were also unable to compare self-reported survey responses with EHR data, so we are unable to assess accuracy of patient self-reports of cancer type, cancer stage at diagnosis, and dates of first visit/test and diagnosis used to calculate the diagnostic interval. Patients who reported their cancer was diagnosed through symptomatic presentations or at later stages tended to report worse care experiences than patients whose cancer was detected through screening or incidental findings, or at earlier stages of disease, aligning with previous work [46]. These reported differences in care experiences by presentation and cancer stage may be due, in part, to factors associated with presentation and stage, such as socioeconomic status. The survey also does not account for possible hindsight or outcome bias on the part of the respondents. We chose not to risk-adjust the measures when stratifying by respondent subgroups due to our small sample size.

Lastly, this survey’s purview is restricted to the period between when the patient is first seen by a health care professional and the final diagnosis (diagnostic interval). Important delays may also happen during the appraisal interval (when patients are deciding whether to seek care) and help-seeking interval (when patients have decided to seek care but have not yet received it). We will explore this in future surveys.

## Conclusions

Timely and accurate diagnosis of cancer remains an important target for quality measurement and quality improvement. In our study, most respondents reported easy access and excellent communication with both usual care providers and specialists, ease of access to tests and results, and rated overall care highly, in line with previous work [47], but results also indicated numerous areas with substantial room for improvement.

Our study establishes internal consistency, reliability, and construct validity of patient-reported measures assessing aspects of timely diagnosis of cancer. These measures can be used to identify targets for quality improvement and complement other measures assessing timely diagnosis, such as current claims- and EHR-based measures [48], 49] as well as emerging approaches, such as the use of artificial intelligence to rapidly analyze clinical notes, conversations between clinicians and patients, and stories on social media. In the pursuit of diagnostic excellence for cancer in the US, patient-reported measures allow us to learn about processes and perspectives that would otherwise be unobservable.

## Supplementary Material

Supplementary Material

Supplementary Material
