# Supplementary material for: Surveying patients to assess timeliness of cancer diagnosis
Source: Diagnosis (Berl). 2025 Oct 27;13(2):300–11. doi: 10.1515/dx-2024-0203 (PMC13075801; doi:10.1515/dx-2024-0203)
Supplement: Supplementary file 1 [file j_dx-2024-0203_suppl_001.pdf]

Draft

Survey About Health Care Leading Up to a Cancer Diagnosis

This survey will ask questions about your health care. When answering, please think about care received at Cedars-Sinai and anywhere else you have received health care recently.

The survey will only take about 20 minutes to complete. You can ask a family member or friend for help with this survey or ask them to complete the survey for you. If you are completing this survey on behalf of someone else, please remember that survey questions refer to the patient and their care.

Introduction

1. **In the last 12 months, has a doctor, nurse, or other health care professional told you that you definitely have cancer?**
   - ☐ Yes
   - ☐ No → **Go to Question 29**
   - ☐ Don't know/not sure → **Go to Question 29**

2. **In the last 12 months, what kind of cancer did this health care professional say you have? Choose one.**
   - ☐ Bladder
   - ☐ Breast
   - ☐ Colon and Rectal (Colorectal)
   - ☐ Endometrial
   - ☐ Kidney
   - ☐ Leukemia
   - ☐ Liver
   - ☐ Lung
   - ☐ Melanoma
   - ☐ Non-Hodgkin Lymphoma
   - ☐ Pancreatic
   - ☐ Prostate
   - ☐ Thyroid
   - ☐ Other (please print): ____________

Draft

3. What stage was this cancer when you were diagnosed?

- ☐ Stage 0 (sometimes called "in situ")
- ☐ Stage 1
- ☐ Stage 2
- ☐ Stage 3
- ☐ Stage 4 (sometimes called "metastatic")
- ☐ Don't know/not sure

The Time Leading Up To Your Cancer Diagnosis

4. We are trying to learn about the *time leading up to your cancer diagnosis*. This time period may have been very short or could have gone on for weeks or months. At the beginning of the time leading up to your cancer diagnosis, you may have gone to a health care professional because you had symptoms, or you may not have had symptoms, but went for a regular check-up, screening, or some other test that turned out to be related to your cancer.

What was the <u>very first thing</u> that happened that led to your cancer diagnosis? Choose one.

- ☐ I saw a health care professional because I had symptoms (examples: a lump, pain, or unintended weight loss)
- ☐ I saw a health care professional for a check-up and they noticed something concerning
- ☐ I went for a cancer screening test (examples: mammogram, colonoscopy, Pap smear, prostate-specific antigen [PSA] test, or fecal immunochemical test [FIT])
- ☐ I went for a test for another reason (examples: routine blood test, x-ray after an injury)
- ☐ None of the above
- ☐ Don't know/not sure
- ☐ Other (please print): ______

5. What was the date that you saw this health care professional or had the test you mentioned in Question 4? If you cannot remember the exact date, please provide as much information as you can.

☐☐ - ☐☐ - ☐☐☐☐

Month Day Year

6. What was the date of your cancer diagnosis? This is the date you were told you <u>definitely have</u> cancer. If you cannot remember the exact date, please provide as much information as you can.

☐☐ - ☐☐ - ☐☐☐☐

Month Day Year

This survey asks about the *Time Leading Up to Your Cancer Diagnosis*. This is the time starting with all the care you received on <u>this first date</u> and <u>ending with your diagnosis</u> on the second date.

7. ***During the time leading up to your cancer diagnosis*, approximately how many times did you see a health care professional?**

☐ 0

☐ 1

☐ 2

☐ 3 - 4 times

☐ 5 or more times

☐ Don’t know/not sure

8. Do you think your health care professionals could have diagnosed your cancer much sooner than they did?

☐ Yes

☐ No

☐ Don't know/not sure

Care From Your Usual Health Care Professionals

The rest of this survey asks about the care you received in *the time leading up to your cancer diagnosis*. We are NOT asking about the care or treatment you received after your cancer diagnosis.

9. ***BEFORE the time leading up to your cancer diagnosis*, did you have any usual health care professionals such as doctors, clinics, health centers, or other places that you went to if you were sick or needed advice about your health? This could include a primary care doctor, a family doctor, an ob/gyn, dentist, or other professional that you usually saw for your health care.**

☐ Yes

☐ No → **Go to Question 17**

☐ Don't know/not sure → **Go to Question 17**

10. ***During the time leading up to your cancer diagnosis*, did you ever see or talk with any of your usual health care professionals?**

☐ Yes

☐ No → **Go to Question 17**

☐ Don't know/not sure → **Go to Question 17**

11. ***During the time leading up to your cancer diagnosis*****, how often were you able to see your <u>usual health care professional(s)</u> as soon as you needed?**

- ☐ Never
- ☐ Sometimes
- ☐ Usually
- ☐ Always → **Go to Question 13**
- ☐ Don't know/not sure

12. Which of the following prevented you from seeing your <u>usual health care professional(s)</u> as soon as you needed? Check all that apply.

- ☐ I had trouble figuring out how to schedule an appointment
- ☐ I had to wait a long time for an available appointment
- ☐ My usual health care professional(s) was far away or hard to get to
- ☐ I had trouble getting time off work or away from my family responsibilities
- ☐ I was worried about how much it would cost
- ☐ I had trouble getting my insurance to cover it
- ☐ I did not have health insurance or some other kind of health care plan
- ☐ Other (please print):

13. ***During the time leading up to your cancer diagnosis*****, how often did your <u>usual health care professional(s)</u> listen carefully to you?**

- ☐ Never
- ☐ Sometimes
- ☐ Usually
- ☐ Always
- ☐ Don't know/not sure

14. ***During the time leading up to your cancer diagnosis*****, how often did your <u>usual health care professional(s)</u> take your concerns seriously?**

- ☐ Never
- ☐ Sometimes
- ☐ Usually
- ☐ Always
- ☐ Don't know/not sure

Draft

15. ***During the time leading up to your cancer diagnosis*, how often did your <u>usual health care professional(s)</u> treat you with dignity and respect?**

- ☐ Never
- ☐ Sometimes
- ☐ Usually
- ☐ Always
- ☐ Don't know/not sure

16. Specialists are doctors like oncologists, surgeons, and other doctors who specialize in one area of health care.

***During the time leading up to your cancer diagnosis*, did your <u>usual health care professional(s)</u> refer you to another health care professional who is a specialist to help determine if you had cancer?**

- ☐ Yes
- ☐ No
- ☐ Don't know/not sure

Getting Care From Specialists

17. ***During the time leading up to your cancer diagnosis*, did you actually see any specialist(s) to help determine if you had cancer? Specialists are doctors like oncologists, surgeons, and other doctors who specialize in one area of health care.**

- ☐ Yes
- ☐ No → **Go to Question 24**
- ☐ Don't know/not sure → **Go to Question 24**

18. What was the date that you first saw the specialist(s)? If you cannot remember the exact date, please provide as much information as you can.

☐☐ - ☐☐ - ☐☐☐☐

Month Day Year

19. If you saw more than one specialist *during the time leading up to your cancer diagnosis*, please think of all specialists when answering the rest of the questions in this section.

***During the time leading up to your cancer diagnosis*, how often were you able to see the specialist(s) as soon as you needed?**

- ☐ Never
- ☐ Sometimes
- ☐ Usually
- ☐ Always → **Go to Question 21**
- ☐ Don't know/not sure

20. Which of the following prevented you from seeing the specialist(s) as soon as you needed? Check all that apply.

- ☐ I was not referred to a specialist quickly enough
- ☐ I had trouble figuring out how to schedule an appointment
- ☐ I had to wait a long time for an available appointment
- ☐ The specialist was far away or hard to get to
- ☐ I had trouble getting time off work or away from my family responsibilities
- ☐ I was worried about how much it would cost
- ☐ I had trouble getting my insurance to cover it
- ☐ I did not have health insurance or some other kind of health care plan
- ☐ Other (please specify): ______

21. *During the time leading up to your cancer diagnosis*, how often did the specialist(s) you saw listen carefully to you?

- ☐ Never
- ☐ Sometimes
- ☐ Usually
- ☐ Always
- ☐ Don't know/not sure

22. *During the time leading up to your cancer diagnosis*, how often did the specialist(s) you saw take your concerns seriously?

- ☐ Never
- ☐ Sometimes
- ☐ Usually
- ☐ Always
- ☐ Don't know/not sure

Draft

23. ***During the time leading up to your cancer diagnosis*, how often did the specialist(s) you saw treat you with dignity and respect?**

☐ Never

☐ Sometimes

☐ Usually

☐ Always

☐ Don't know/not sure

Other Care

24. ***During the time leading up to your cancer diagnosis*, how often was it easy to get the tests, x-rays, or scans you needed?**

☐ Never

☐ Sometimes

☐ Usually

☐ Always

☐ Don't know/not sure

25. ***During the time leading up to your cancer diagnosis*, when someone ordered a test, x-ray, or scan for you, how often did someone follow up to give you those results?**

☐ Never

☐ Sometimes

☐ Usually

☐ Always

☐ Don't know/not sure

26. An interpreter is someone who helps you talk with others who do not speak your language. Interpreters can include health care staff or telephone interpreters. *During the time leading up to your cancer diagnosis*, was there any time when you wanted an interpreter?

☐ Yes

☐ No → **Go to Question 28**

27. ***During the time leading up to your cancer diagnosis*, when you wanted an interpreter to speak with your health care professional(s), how often did you get one?**

☐ Never

☐ Sometimes

☐ Usually

☐ Always

☐ Don't know/not sure

Draft

Overall Experience

28. Using any number from 0 to 10, where 0 is the worst care possible and 10 is the best care possible, what number would you use to rate the care you received from <u>all</u> your health care professionals *during the time leading up to your cancer diagnosis*?

- ☐ 0 Worst care possible
- ☐ 1
- ☐ 2
- ☐ 3
- ☐ 4
- ☐ 5
- ☐ 6
- ☐ 7
- ☐ 8
- ☐ 9
- ☐ 10 Best care possible
- ☐ Don't know/not sure

About You

29. *During the time leading up to your cancer diagnosis*, did you have any type of health insurance or some other type of health care plan?

- ☐ Yes, for all of the time
- ☐ Yes, for some of the time
- ☐ No

30. How old are you? If you are 90 or older, please put "90" in the box.

☐☐

31. What is the highest grade or level of school that you have completed?

- ☐ 8th grade or less
- ☐ Some high school but did not graduate
- ☐ High school graduate or GED
- ☐ Some college or 2-year degree
- ☐ 4-year college graduate
- ☐ More than 4-year college degree

Draft

32. How do you describe yourself?

- ☐ Female
- ☐ Male
- ☐ Trans female/trans woman
- ☐ Trans male/trans man
- ☐ Genderqueer/gender non-conforming
- ☐ Different identity (please state): ____________

33. Are you of Hispanic or Latino origin or descent?

- ☐ Yes, Hispanic or Latino
- ☐ No, not Hispanic or Latino

34. What is your race? Please choose one or more.

- ☐ White
- ☐ Black or African American
- ☐ Asian
- ☐ Native Hawaiian or other Pacific Islander
- ☐ American Indian or Alaska Native
- ☐ Other (please specify): ____________

35. What language do you mainly speak at home?

- ☐ English
- ☐ Spanish
- ☐ Some other language (please print): ____________

36. Did someone help you complete this survey? Check all that apply.

- ☐ No, no one helped me complete this survey → **Go to Question 38**
- ☐ Yes, they read the questions to me
- ☐ Yes, they wrote down the answers I gave
- ☐ Yes, they answered the questions for me
- ☐ Yes, they translated the questions into my language
- ☐ Yes, they helped in some other way

Draft

37. For what reason did someone help you complete this survey? Check all that apply.

- ☐ Too sick
- ☐ Trouble with memory
- ☐ Trouble seeing or reading
- ☐ Hard of hearing
- ☐ Do not understand English
- ☐ Wanted someone else to complete the survey
- ☐ Patient is in a coma
- ☐ Patient has passed away
- ☐ Some other reason (please print):

Other Thoughts

38. In thinking about your experiences during the time leading up to your cancer diagnosis, what went well? Please tell us about those experiences.

39. In thinking about your experiences during the time leading up to your cancer diagnosis, what do you wish had gone differently? Please tell us about those experiences.

Draft
