# Supplementary material for: Surveying patients to assess timeliness of cancer diagnosis
Source: Diagnosis (Berl). 2025 Oct 27;13(2):300–11. doi: 10.1515/dx-2024-0203 (PMC13075801; doi:10.1515/dx-2024-0203)
Supplement: Supplementary file 2 [file j_dx-2024-0203_suppl_002.pdf]

Supplemental Material 1

Appendix Table A.1: Characteristics of n=1,174 Measure Denominator Eligible Patients

**Characteristic**	**n (%)**
Sex	
Female	664 (56.6%)
Male	510 (43.4%)
Race	
American Indian or Alaska Native	4 (0.3%)
Asian	138 (11.8%)
Black/African American	108 (9.2%)
Native Hawaiian or Other Pacific Islander	6 (0.5%)
White	787 (67.0%)
Other	101 (8.6%)
Unknown/Missing	30 (2.6%)
Ethnicity	
Non-Hispanic	974 (83.0%)
Hispanic	163 (13.9%)
Unknown/Missing	37 (3.1%)
Age	
18-39 years	101 (8.6%)
40-64 years	455 (38.8%)
65-80 years	490 (41.7%)
>80 years	128 (10.9%)
Primary ICD-10 Diagnosis Code	
C00-C14: Malignant neoplasms of lip, oral cavity and pharynx	13 (1.1%)
C15-C26: Malignant neoplasms of digestive organs	217 (18.5%)
C30-C39: Malignant neoplasms of respiratory and intrathoracic organs	71 (6.0%)
C40-C41: Malignant neoplasms of bone and articular cartilage	8 (0.7%)
C43: Melanoma and other malignant neoplasms of skin	12 (1.0%)
C45-C49: Malignant neoplasm of mesothelial and soft tissue	42 (3.6%)
C50: Malignant neoplasms of breast	307 (26.2%)
C51-C58: Malignant neoplasms of female genital organs	56 (4.8%)
C60-C63: Malignant neoplasms of male genital organs	122 (10.4%)
C64-C68: Malignant neoplasms of urinary tract	69 (5.9%)
C69-C72: Malignant neoplasms of eye, brain and other parts of central nervous system	38 (3.2%)
C73-C75: Malignant neoplasms of thyroid and other endocrine glands	40 (3.4%)
C76-C80: Malignant neoplasms of ill-defined, other secondary and unspecified sites	51 (4.3%)
C7A: Malignant neuroendocrine tumors	13 (1.1%)
C81-C95: Malignant neoplasms of lymphoid, hematopoietic and related tissue	115 (9.8%)
Geographic Location	
Zip Code in Los Angeles County	936 (79.7%)
Other Zip Code	238 (20.3%)
Contact Method	
Email and Postal Address Available	1100 (93.7%)
Only Postal Address Available	74 (6.3%)

Notes: Characteristics obtained from electronic health records

Appendix Table A.2: Distribution of Responses to Evaluative Survey Items.

**Measure Type**<br>**Measure**<br>Item<br>Response options	n (%)
**Measures of Aspects of Care**	
**Getting Timely Usual Care**	
Able to see usual health care professionals as soon as needed	
Never	11 (5.6%)
Sometimes	22 (11.3%)
Usually	54 (27.7%)
Always	103 (52.8%)
Don't know/not sure	5 (2.6%)
**Communication with Usual Care Professionals**	
Listen carefully to you	
Never	8 (4.1%)
Sometimes	15 (7.6%)
Usually	32 (16.2%)
Always	138 (70.1%)
Don't know/not sure	4 (2.0%)
Take concerns seriously	
Never	12 (6.2%)
Sometimes	12 (6.2%)
Usually	27 (13.9%)
Always	141 (72.7%)
Don't know/not sure	2 (1.0%)
Treat with dignity and respect	
Never	2 (1.0%)
Sometimes	6 (3.1%)
Usually	20 (10.3%)
Always	164 (84.1%)
Don't know/not sure	3 (1.5%)
**Getting Timely Specialty Care**	
Able to see usual health care professionals as soon as needed	
Never	6 (3.2%)
Sometimes	16 (8.6%)
Usually	43 (23.1%)
Always	115 (61.8%)
Don't know/not sure	6 (3.2%)
**Communication with Specialists**	
Listen carefully to you	
Never	1 (0.5%)
Sometimes	6 (3.1%)
Usually	21 (10.9%)
Always	162 (84.4%)
Don't know/not sure	2 (1.0%)
Take concerns seriously	
Never	1 (0.5%)

Sometimes	7 (3.6%)
Usually	18 (9.4%)
Always	164 (85.4%)
Don’t know/not sure	2 (1.0%)
Treat with dignity and respect	
Never	0 (0.0%)
Sometimes	5 (2.6%)
Usually	10 (5.2%)
Always	176 (91.7%)
Don’t know/not sure	1 (0.5%)
**Getting Tests and Results**	
Easy to get tests, x-rays, or scans	
Never	16 (5.9%)
Sometimes	26 (9.6%)
Usually	57 (21%)
Always	168 (61.8%)
Don’t know/Not sure	5 (1.8%)
Results communicated	
Never	6 (2.2%)
Sometimes	26 (9.6%)
Usually	44 (16.3%)
Always	191 (70.7%)
Don’t know/Not sure	3 (1.1%)
**Overall Assessment Measures**	
**Overall rating of care during diagnostic interval**	
0-6	28 (10.3%)
7-8	42 (15.5%)
9-10	198 (73.1%)
Don’t know/not sure	3 (1.1%)
**Perceived delay in cancer diagnosis (think health care professionals could have diagnosed cancer much sooner than they did)**	
Yes	139 (50.7%)
No	78 (28.5%)
Don’t know/not sure	57 (20.8%)

Appendix Table A.3: Top-Box Scores for Evaluative Survey, Overall and for Subgroups of Interest.

		Initial Presentation (n=266)				Cancer Stage at Diagnosis (n=186)			
	All (n=276)	Screening test (n=60)	Incidental finding (n=84)	Symptomatic (n=122)	p-value	0-1 (n=78)	2-3 (n=67)	4 (n=41)	p-value
**Measures of Aspects of Care**									
Getting Timely Usual Care	54%	66%	53%	47%	0.144	53%	57%	44%	0.551
Communication with Usual Care Professionals	76%	88%	76%	68%	0.014	85%	76%	49%	0.000
1. Listen carefully	72%	84%	66%	66%	0.061	80%	74%	52%	0.028
2. Take concerns seriously	73%	87%	74%	64%	0.024	82%	73%	46%	0.003
3. Dignity and respect	85%	98%	89%	77%	0.005	95%	83%	52%	<0.001
Getting Timely Specialty Care	64%	70%	58%	64%	0.447	75%	54%	48%	0.030
Communication with Specialists	88%	98%	87%	83%	0.026	89%	86%	94%	0.475
1. Listen carefully	85%	97%	82%	82%	0.059	83%	83%	91%	0.659
2. Take concerns seriously	86%	97%	84%	83%	0.081	87%	85%	95%	0.445
3. Dignity and respect	92%	100%	95%	86%	0.013	96%	89%	95%	0.259
Getting Tests and Results	67%	68%	67%	66%	0.975	69%	68%	62%	0.630
1. Easy to get tests, x-rays, or scans	63%	67%	63%	60%	0.646	69%	65%	54%	0.282
2. Results communicated	72%	69%	70%	74%	0.776	68%	73%	73%	0.798
**Overall Assessment Measures**									
Overall rating of care during diagnostic interval (9-10/10)	74%	83%	69%	72%	0.150	83%	73%	51%	0.002
No perceived delay in cancer diagnosis	64%	75%	66%	57%	0.113	71%	60%	52%	0.133

Top-box scores classify the response(s) indicating the best experiences as 100 and all other responses as 0 (e.g., ‘‘always’’ = 100; all other responses = 0; rating of 9 or 10 out of 10 = 100; all other responses = 0). We tested statistical significance of group comparison for initial presentation and cancer stage diagnosis using ANOVA tests for composite measures, and Chi-square tests for other measures.

Appendix Table A.4. Association of Quality Measure Top-Box Scores with Overall Ratings of Care, Number of Visits During Diagnostic Interval, Diagnostic Interval Less than Days, and Perceived Timeliness of Cancer Diagnosis.

	Overall Rating (0=worst care possible, 10=best)	Number of visits during diagnostic interval	Diagnostic interval <=30 days	Perceived Timeliness of Cancer Diagnosis
	Unstandardized Regression Coefficients [95% CI]		Odds Ratio [95% CI]	
Models Including All Measures Applicable to Patients Receiving Specialty Care				
Getting Timely Usual Care	0.08 [-0.40, 0.57]	0.26 [-0.15, 0.66]	1.21 [0.53, 2.77]	0.96 [0.35, 2.68]
Communication with Usual Care Professionals	1.55*** [0.76, 2.35]	-0.04 [-0.71, 0.63]	0.45 [0.11, 1.80]	7.76* [1.44, 41.80]
Getting Timely Specialty Care	0.33 [-0.25, 0.90]	0.10 [-0.38, 0.58]	1.42 [0.53, 3.80]	1.02 [0.32, 3.28]
Communication with Specialists	1.80*** [0.91, 2.69]	-0.57 [-1.32, 0.18]	1.04 [0.22, 5.03]	3.05 [0.51, 18.34]
Getting Tests and Results	1.05** [0.32, 1.78]	-0.02 [-0.65, 0.61]	3.54 [0.99, 12.62]	2.70 [0.64, 11.42]
Models Including Measures Applicable to All Patients				
Getting Timely Usual Care	0.10 [-0.40, 0.59]	0.34 [-0.01, 0.70]	1.08 [0.53, 2.19]	1.34 [0.59, 3.07]
Communication with Usual Care Professionals	2.80*** [2.08, 3.52]	-0.60* [-1.13, -0.08]	0.62 [0.22, 1.76]	9.90*** [2.66, 36.75]
Getting Tests and Results	1.56*** [0.93, 2.19]	-0.03 [-0.49, 0.42]	4.14** [1.63, 10.48]	3.42* [1.14, 10.31]

* p<0.05; ** p<0.01; *** p<0.001

"Models Including All Measures Applicable to Patients Receiving Specialty Care" assesses the unique contribution of each measure in models that included all five measures; these analyses were conducted on the subset of respondents that received care from specialists.

"Models Including Measures Applicable to All Patients" assess the unique contribution of each measure in models that includes the three measures applicable to all respondents (i.e., regardless of whether they received care from specialists).

All models restrict to respondents with non-missing scores for the model's respective outcome and predictors.
